# Objections to the Theories of Franklin, Dufay, or Ampere, with an Effort to Explain Electrical Phenomena by the Statical or Undulatory Polarization of Omnipresent, Ethereal, or Ethereo-ponderable Matter*Read before the Academy of Natural Sciences, and although ordered to be published in the forthcoming volume, has been excluded by matter having priority of date. Hence, to avoid procrastination-with permission from the Academy-it has been communicated to the obliging Editor of this work.This commnnication might have been offered to the Journal in which those affiliated with the University usually make their communications, were it not that my friend, the editor, to an inquiry replied, that “*nothing which is not directly applied to medicine*” would be published in that channel.

**Published:** 1847-12

**Authors:** Robert Hare

**Affiliations:** Emeritus Professor of Chemistry in the University of Pennsylvania


### MEDICAL EXAMINER,

AND

### RECORD OF MEDICAL SCIENCE

NEW SERIES.—No. XXXVI.—DECEMBER, 1847.

### ORIGINAL COMMUNICATIONS.

*Objections to the Theories of Franklin, Dufay, or Ampere, with an effort to explain electrical phenomena by the statical or undulatory polarization of omnipresent, ethereal, or ethereo-ponderable matter.** By ROBERT HARE, M. D., Emeritus Professor of Chemistry in the University of Pennsylvania.

1. It appears, from the experiments of Wheatstone, that the discharge of a Leyden jar, by means of a copper wire, takes place within a time so small, that were the transfer of a fluid from the positive to the negative surface requisite for its accomplishment, a current having a velocity exceeding two hundred thousand miles in a second would be necessary.

2. The only causes for the velocity of an electric current, according to Franklin, are the repulsion between the particles of the electric fluid, of which it has been assumed to consist, and

*Read before the Academy of Natural Sciences, and although ordered to be published in the forthcoming volume, has been excluded by matter having priority of date. Hence, to avoid procrastination—with permission from the Academy—it has been communicated to the obliging Editor of this work.

This commnnication might have been offered to the Journal in which those affiliated with the University usually make their communications, were it not that my friend, the editor, to an inquiry replied, that *"nothing which is not directly applied to medicine"* would be published in that channel.

the attraction between those particles and other matter. These forces are alleged to concur in distributing the supposed fluid throughout space; whether otherwise void, or partially pre-occupied by conducting solids or fluids. Hence, when between two or more spaces, surfaces, or conducting masses, there is an unequal distribution of the electric fluid, the equilibrium is restored whenever a communication is opened, by means of a sufficiently conducting medium. Agreeably to this view of the subject, there seems to be a resemblance between the supposed effort of the electrical fluid to attain a state of equable diffusion, and that which would exist in the case of a gas confined in adjoining receivers, so as to be more dense within one than within the other; for, however the subtility of the supposed electric fluid may exceed that of any gas, there seems to be an analogy as respects the processes of diffusion which must prevail. But on the escape of any elastic fluid from a cavity, within which it may be condensed, evidently there must be a diminution of density, and of the consequent velocity of efflux, proportionally as the quantity left in the cavity diminishes, so that the latter portions would move with extreme slowness. Far from taking place in an analogous manner, electrical discharges are effected with an extreme suddenness, the whole of the redundancy being discharged at once in a mode more like the flight of a bullet, projected with infinite velocity, than that of a jet varying in celerity from a maximum to a minimum.

3. So far, in fact, is an electrical discharge from displaying the features which belong to the reaction of a condensed elastic fluid, that agreeably to the observations of Henry, the result is more like the vibrations of a spring, which, in striving to regain its normal position, goes beyond it. The first discharge between the surfaces of a Leyden jar is not productive of a perfect equilibrium. The wave-like transfer of different polarities goes beyond the point of reciprocal neutralization, producing a state, to a small extent, the opposite of that at first existing; and hence a refluent discharge ensues, opposite in direction to the primary one. But even this does not produce an equilibrium, so that a third effort is made. These alternate discharges were detected by means of the magnetism imparted to needles exposed, in helices, to the consequent current, more properly called polarizing undulations or waves.

4. Supposing one or more rows of electrical particles, forming such a filament of electricity as must occupy the space within a wire of great length, to be made the medium of discharge to a Leyden jar; agreeably to the hypothesis of one fluid, the electrical filament must be attracted at one end of the wire, and propelled at the other, as soon as its terminations are brought into due communication with the coatings of the jar. Yet the influence of the oppositely charged surfaces of the jar cannot be conceived to extend to those portions of the electricity which are remote from the points of contact, until they be reached by a succession of vibrations. Hence it is inconceivable that every particle in the filament of electric matter can be made at the same time to move, so as to constitute a current having the necessary velocity and volume to transfer, instantaneously, the electricity requisite to constitute a charge. Even the transmission of the impulses, in such an infinitesimal of time, seems to be inconceivable.

5. In reply to these objections, it has been urged by the Franklinians, that a conductor being replete with electricity, as soon as this fluid should be moved at one end, it ought to move at the other. This might be true of an incompressible fluid, but could not hold good were it elastic. A bell wire moves at both ends when pulled only at one; but this would not ensue were a cord of gum elastic substituted for the wire.

6. But if the flow of one fluid, with the enormous velocity inferred, be difficult to conceive, still more must it be incomprehensible that two fluids can rush with similar celerity, from each surface of the jar, in opposite directions, through the narrow channel afforded by a wire; especially as they are alleged to exercise an intense affinity; so that it is only by a series of decompositions and recompositions that they can pass each other. That agreeably to the theory of Dufay, equivalent portions of the resinous and vitreous fluids must exchange places during an electrical discharge, will appear evident from the following considerations:

7. One surface being redundant with vitreous and deficient commensurately of resinous electricity, and the other redundant with the resinous and deficient of the vitreous fluid, it is inevitable that to restore the equilibrium, there must be a simultaneous transfer of each redundancy to the surface wherein there is a deficiency of it to be supplied. If after decomposing a large portion of the neutral compound, previously existing on the surfaces of the jar, and transferring the ingredients severally in opposite directions, so as to cause each to exist in excess upon the surface assigned to it, were the redundancies, thus originated, to be neutralized by meeting in the discharging rod, neither surface could recover its quota of the electrical ingredient of which it must have been deprived agreeably to the premises.

8. This calls to mind the fact, that no evidence has been adduced of the existence of any tertium quid, arising from the union of the supposed electricities founded on any property displayed by their resulting combination in the neutral state. It must, if it exists, constitute an anomalous matter, destitute of all properties, and of the existence of which we have no evidence, besides that founded on the appearance and disappearance of its alleged ingredients.

9. But however plausibly the discharges consequent to making a conducting communication from one electrified mass or surface to another mass or surface in an opposite state, may be ascribed to accumulations either of one or of two fluids; neither, according to one theory nor the other, is it possible to account satisfactorily for the stationary magnetism with which steel may be endowed, nor the transitory magnetism, or dynamic power of induction, acquired by wires transmitting galvanic discharges.

10. For the most plausible effort which has been made for the purpose of reconciling the phenomena of electro-magnetism with the theory of two fluids, or with that of one fluid, so far as these theorie are convertible, we are indebted to Ampere.

11. According to the hypothesis advanced by this eminent philosopher, the difference between a magnetised and an electrified body, is not attributable to any diversity in the imponderable matter to which their properties are respectively due, but to a difference in the actual state or distribution of that matter. Statical polarity is the consequence of the unequal distribution of the two electric fluids whose existence he assumes; while magnetical polarity is the consequence merely of the motion of those fluids; which, in magnets, are supposed to gyrate in opposite directions about each particle of the mass.

These gyrations are conceived to take place only in planes at right angles to the axis of the magnet; so that, in a straight magnet, the planes of the orbits must be parallel to each other.*

12. The aggregate effect of all the minute vortices of the electrical fluids, in any one plane, bounded by the lateral surfaces of the magnet, is equivalent externally to one vortex, since, in either case, every electric particle on that surface will so move as to describe tangents to a circle drawn about the axis of the magnet. When the electrical vortices of the pole of one magnet, conflict in their direction with those of another, as when similar magnetic poles are approximated, repulsion ensues; but if the vortices are coincident in direction, as when dissimilar poles are near, attraction takes place. When a current through a galvanized wire concurs in direction with the magnetic vortices, as above described, attraction ensues; repulsion resulting when it does not so concur. Hence, the magnet, if moveable, will strive to assume a position, in which its electrical currents will not conflict with those of the wire on one side more than on the other: also, the wire, if moveable, will tend so to arrange itself as to produce the same result, which can arrive only when the needle is

* The words gyration, vortex and whirl, are considered as synonymous, and alternately used only to avoid monotony.

at right angles to the wire, and its sides consequently equi-distant therefrom.

13. Electric currents will produce magnetic vortices, and reciprocally, magnetic vortices will produce electric currents. Hence the magnetism imparted to iron by galvanized spirals, and the Faradian currents produced by magnetized iron within spirals not galvanized.

14. Ampere's theory has, in a high degree, the usual fault of substituting one mystery for another; but, on the other hand, it has, in an equally high extent, the only merit to which any theory can make an indisputable claim: I mean, that of associating facts so as to make them more easy to comprehend and to remember, enabling us, by analogy, to forsee results, and thus affording a clue in our investigations. Evidently, the author of this theory was guided by it, in his highly interesting and instructive contrivances; and Professor Henry ascribes his success in improving the electro-magnet, to the theoretic clue which he had received from Ampere.

15. Nevertheless, the postulates on which this Amperian hypothesis is founded, appear to me unreasonable. They require us to concede that about every atom of a permanent magnet a process is going on, analogous to that generally admitted to exist in a galvanic circuit, where two fluids pass each other in a common channel by a series of decompositions and recompositions. In the galvanic circuit this process is sustained by chemical reaction, but in magnets having no co-enduring reagency to sustain it, the heterogenous constituents of an imaginary electrical tertium quid, are presumed to be perpetually separating in order to recombine.

16. In cases of complex affinity, where four particles, A B C D are united into two compounds A B, C D, it is easy to conceive that, in obedience to a stronger affinity, A shall combine with B, and C with D: but, without any extraneous agency, wherefore, in any one compound, should a particle A quit one particle B, in order to unite with another particle of the same kind; or wherefore should any one, B, quit one A, in order to combine with another A.

17. That such a process should ensue by a species of catalytic agency exercised by a magnet or galvanized conductor, while in proximity, were difficult to understand; but to me it is utterly unintelligible that the transient influence of such a cause, should be productive of permanent gyrations.

18. Moreover, it is inconceivable that the particles of any matter should, as required by this hypothesis, *merely by being put into motion*, acquire a power of reciprocal repulsion, or attraction, of which it were otherwise destitute. The vortices being assumed to take place about each atom, cannot severally occupy an area of greater diameter than can exist between the centres of any two atoms. Of course, the gyratory force exercised about the surface of a magnet by the aggregate movements of the vortices, cannot extend beyond the surface more than half the diameter of one of the minute areas of gyration alluded to. Wherefore, then, do these gyrations, when similar in direction, from their concurrence approach each other; when dissimilar in direction move asunder, even when situated comparatively at a great distance?

19. I should consider Ampere's theory as more reasonable, were it founded upon the existence of one fluid; since, in that case, vortices might be imagined without the necessity of supposing an endless and unaccountable separation and reunion of two sets of particles; not only devoid of any property capable of sustaining their alleged opposite gyrations, but actually endowed with an intense reciprocal attraction which must render such gyrations impossible.

But even if grounded on the idea of one fluid, this celebrated hypothesis does not seem to me to account for the phenomena which it was intended to explain. If distinct portions of any fluid do not attract or repel each other when at rest, wherefore should they either attract or repel each other when in motion. Evidently mere motion can generate neither attraction nor repulsion. Bodies subjected to a projectile force gravitate with the same intensity and descend in obedience to terrestrial attraction, through the same perpendicular distance, whether moving with the celerity of a cannon ball, or a planet, or undergoing no impulse excepting those arising from their own unresisted weights.

20. The objections which are thus shown to be applicable in the case of liquids, of which the neighbouring particles are destitute of the reaction requisite to produce the phenomena requiring explanation, must operate with still greater force where ethereal fluids are in question, of which the properties are positively irreconcileable with the phenomena. According both to Franklin and Dufay, bodies, when similarly electrified, should repel each other; yet in point of fact, collateral wires, when subjected to similar voltaic discharges, and of course similarly electrified, become reciprocally attractive, while such wires, when dissimilarly electrified by currents which are not analogous, become reciprocally repulsive.

21. Agreeably to Ampere, an iron bar, situated within a coil of wire subjected to a galvanic current, is magnetized, because the current, in the wire, is productive of an electrical whirlpool about every particle of the metal. When the iron is soft the magnetism, and of course the gyrations of which its magnetism consists by the premises, cease for the most part as soon as the circuit through the coil is broken; but when the iron is in the more rigid state of hardened steel, the gyrations continue for any length of time after the exciting cause has ceased.

22. This theory does not explain wherefore the hardening of the steel should cause the vortical motion to be more difficult to induce yet more lasting when its induction is effected. Evidently the metallic particles must take some part in the process; since it is dependent, for its existence and endurance, upon their nature and their state. Yet no function is assigned to these particles. In fact, it is inconceivable, either that they can participate in, or contribute to the supposed vortical motion.

23. The electrical fluid in an iron bar, cannot form a vortex about each particle, all the vortices turning in one direction, without a conflict between those which are contiguous. In order not to conflict with each other, the alternate vortices would have to turn in different directions, like interlocking cog-wheels in machinery. But in that case, if magnetism be due to currents, the magneto-inductive influence of one set would neutralize that of the other. Again, how can a current, excited by a battery in one circuitous conductor, cause, by dynamic induction, a current in the opposite direction, through another conductor parallel to the first, but insulated therefrom? How can a current of quantity in a ribbon coil, give rise to one of intensity in a helix of fine wire?

24. Having stated my objections to the electrical theories heretofore advanced, it may be proper that I should suggest any hypothetical views which may appear to me of a character to amend or to supersede those to which I have objected. But however I may have been emboldened to point out defects which have appeared to me to be inherent in the theories heretofore accredited, I am far from presuming to devise any substitute which will be unobjectionable. I am fully aware that there is an obscurity as respects the nature and mutual influence of chemical affinity, heat, light, electricity, magnetism and vitality, which science can only to a minute extent dispel.

25. The hypothesis which I now deem preferable is so much indebted to the researches and suggestions of Faraday and others, that, were it true, I could claim for myself but a small share of the merit of its origination.

26. That sagacious electrician employs the following language: "*In the long continued course of experimental inquiry, in which I have been engaged, this general result has pressed upon me constantly, namely, the necessity of admitting two forces or directions of force combined with the impossibility of separating these two forces or electricities from each other.*"—*Experimental Researches*, 1163.

27. Subsequently (1244) after citing another proof of the inseparability of the two electric forces, he alleges it *to be another argument in favour of the view that induction and its concomitant phenomena depend upon* a polarity of the particles of matter!

28. Graham, in his Elements, treating of electricity, alleges that the "great discoveries of Faraday have completely altered the aspect of this department of science, and suggests that all electrical phenomena whatever involve the presence of matter."

29. Unless the distinguished author, from whom this quotation is made, intended to restrict the meaning of the word matter to ponderable matter, there was no novelty in the idea that electrical phenomena involve the presence of matter, since the hypotheses of Franklin and Dufay assume the existence of one or more imponderable material fluids. But, on the other hand, if the meaning of the word *matter* is only to comprise that which is ponderable, the allegation is inconsistent with the authority cited. According to the researches of Faraday, there is an enormous electrical power in metals, and according to his speculations, such powers must be considered as imponderable material principles, pervading the space within which they prevail, independently of any ponderable atom acting as a basis for material properties; the existence of such atoms being represented as questionable.

*Grounds on which a Theory may be advanced.*

30. The existence of two distinct electro-polar forces acting in opposite directions, and necessarily connate and co-existent, yet capable of reciprocal neutralization, agreeably to the authority of Faraday and others.

31. General polarity of matter in assuming the solid state, as displayed during crystallization and vegetation of salts; also as made evident by Faraday's late researches, and the experiments and observations of Hunt.

32. That four hundred and thirty atoms which form a cube of potassium in the metallic state, must occupy nearly six times as much space as the same number of similar atoms fill when existing in a cube of hydrated oxide of potassium of the same size; which, besides seven hundred metallic atoms, must hold seven hundred atoms of hydrogen and fourteen hundred of oxygen, in all two thousand eight hundred atoms; whence it follows there must, in the metallic cube, be *room* for six times as many atoms as it actually holds.*

33. With all due deference, I am of opinion that Faraday has not been consistent in assuming that, agreeably to the Newtonian idea of ponderable atoms, the space in potassium not replete with metal must be vacant; since, according to facts established by his researches, or resulting therefrom, an enormous quantity, both of the causes of heat and of electricity, exists in metals. Moreover, agreeably to his recent speculations, those causes must consist of material, independent, imponderable matter, occupying the whole of the space in which their efficacy is perceptible.*

* Silliman's Am. Jour. Sci., v. 48, p. 247. L. & E. Phil. Mag. and Jour., v. 27, p. 136, v. 25, p. 602.

34. To the evolution of the imponderable matter thus associated, the incandescence of a globule of potassium on contact with water, may be explained, since it is the consequence of the displacement of such matter by the oxygen and hydrogen, which, in replacing it, converts the metal into the hydrated oxide called caustic potash.

The existence both of the causes of electricity and heat in metals, is likewise confirmed by the fact, that the inductive influence of a magnet is sufficient to cause all the phenomena of heat, electrolysis and magnetism, as exemplified by the magneto-electric machine. The existence of the cause of heat in metals is also evident from the ignition of an iron rod when hammered, or the deflagration of wire by the discharge of a Leyden battery.

35. The superiority of metals as electrical conductors, may be the consequence of the pre-eminent abundance of imponderable matter entering into their composition, as alluded to in the case of potassium. (32.)

36. It having been shown that in electrical discharges there cannot reasonably be any transfer of matter, so as to justify the idea of their being the consequence either of one current or of two currents, the only alternative seems to be that the phenomena are due to a progressive affection of the conducting medium, analogous in its mode of propagation to waves, as in the case of liquids, or the aerial or ethereal undulations to which sound and light are ascribed. (1, 2, 3, &c. &c.)

37. The idea intended to be conveyed by the word wave, as applied in common to the undulatory affections above mentioned, and that which is conceived to be the cause of the phenomena usually ascribed to one or more electrical currents, requires only that there should be a state of matter, which, while it may be utterly different from either of those which constitute the waves of water, light or sound, may, nevertheless, like either, pass successively from one portion of a mass to another.

38. The affection thus designated may be reasonably distinguished from other waves, as a *wave of polarization,* since the wire acts, so long as subjected to the reiterated discharges of a voltaic series, as if it were converted into innumerable small magnets, situated like tangents to radii proceeding from its axis.

39. But if a polarizable medium be requisite to electrical discharges, as they pass through a space when devoid of *ponderable* matter, there must be some *imponderable* medium through which they can be effected. Hence we have reason to infer that there is an imponderable matter existing throughout all space, as well as within conductors, which is more or less the medium of the opposite waves essential to electric discharges. Quoting his own language, Davy's experiments led him to consider "that space, (meaning void space,) where there is an appreciable quantity of this matter, (meaning ponderable matter,) is capable of exhibiting electrical phenomena :" also that such phenomena "are produced by a highly subtile fluid or fluids." Moreover, that "it may be assumed, as in the hypothesis of Hooke, Euler, and Huygens, that an ethereal matter susceptible of electrical affections fills all space.*

40. Agreeably to the suggestions above made, all ponderable matter which is liable to be electrified *internally* by electrical discharges, may be considered as consisting of atoms composed of imponderable ethereo-electric particles in a state of combination with ponderable particles, analogous to that which has been supposed to exist between such particles and caloric when causing expansion, liquidity or the aeriform state. Atoms so constituted of ethereal and ponderable particles, may be designated as ethereo-ponderable atoms.

41. A quiescent charge of statical electricity, only affecting the superficies of any ponderable mass with which it may be associated, and having no influence upon the component ethereo-ponderable atoms severally, is not to be ascribed to redundancies or deficiencies of the ethereal matter, but to different states of polarization produced in different sets of the particles of such matter existing about the electrifiable bodies. During the action of an electrical machine, these particles are polarized by the opposite polarities transiently induced in the surfaces subjected to friction; one set of particles going with the electric, the other remaining with the rubber.

42. The particles thus oppositely polarized, severally divide their appropriate polarities with other ethereal matter surrounding the conductors, and this, when insulated, is retained until a further polarization results from the same process. Thus are the ethereo-electric atmospheres respectively surrounding the positive and negative conductors oppositely polarized, and consequently charged to the degree which the machine is competent to induce. Under these circumstances, if a rod be made to form between them a conducting communication, by touching each conductor with one of its ends, the polarities of the ethereo-electric atmospheres by which they are severally surrounded, propagate themselves, by a wave-like process, over the surface of the

* Philosophical Magazine, vol. 60, p. 172. 1822.

conductor, so as to meet midway, and thus produce reciprocal neutralization.

43. In proportion, however, as a wire is small in comparison with the charge, which it may be made the means of neutralizing, the conducting power, seems to be more dependent on the sectional area, and less upon the extent of surface. The reciprocal repulsion of the similarly polarized ethereal particles, must tend always to make them seek the surface, but at the same time, their attraction for the ethereo-ponderable particles composing the wire has the opposite effect, and tends to derange these from their normal polar state of quiescence. In proportion to the degree in which this state is subverted, is the resulting heat, electrolytic power, and electro-magnetic influence. The phenomena last mentioned, are, however, secondary effects consequent to the participation of the ethereo-ponderable matter in the undulations resulting from the statical discharge.

44. Such effects, making allowance for the extreme minuteness of the time occupied by the process, are probably, in all cases, proportional to the degree in which the ponderable matter is effected, up to the point at which it is dissipated by deflagration; but the duration of a statical discharge, being almost infinitely minute for any length of coil which can conveniently be subjected thereto, the electro-magnetic and other effects of a statical discharge are not commensurate with the intensity of the affection of the wire.

45. There is, in fact, this additional reason for the diversity between the electro-magnetic power of a statical discharge, as compared with that of the voltaic series: any wire which is of sufficient length and tenuity to display the maximum power of deflagration by the former, cannot serve for the same purpose in the case of the latter. Moreover, the form of a helix closely wound, so that the coatings may touch, which is that most favourable for the reiteration of the magnetic influence of the circuit upon an iron rod, cannot be adopted in the case of statical discharges, of high intensity, since the proximity of the circumvolutions would enable the ethereal waves, notwithstanding the interposition of cotton or silk, to cross superficially from one to the other, parallel to the axis of the included iron, instead of pursuing the circuitous channel afforded by the helix with the intensity requisite to the polarization of the ponderable atoms.

46. The intensity of the excitement produced by different electrical machines, is estimated to be as the relative lengths of the sparks which proceed from their prime conductors respectively. Admitting that the relative intensity were merely as the length of the spark, not as the square of that length, still there would be an immense difference between the intensity of a voltaic series and that of electrical machines if measured by this test. Large electrical machines, like that at the Polytechnic Institution, London, give sparks at twenty inches and more; while, agreeably to Gassiot's experiments, a Groves battery of 320 pairs, in full power, would not, before contact, give a spark at any distance, however minute. It follows, that, as respects that species of intensity which is indicated by length of sparks, or striking distance, the difference between the electricity of the most powerful voltaic series and electrical machines is not to be represented by any degree of *disparity;* it is rather an utterly discordant feature; it is a trait in which there is an extreme dissimilitude.

47. It should be recollected that the intensity of galvanic action in a series of 320 pairs, excepting the loss from conduction, would be to that of one pair as 320 to one.*

48. We may infer that the undulatory polarization of ethereo-ponderable matter, is the primary, direct, and characteristic effect of galvanic excitement in its more energetic modifications. Yet that by peculiar care in securing insulation, as in the water batteries of Cross and Gassiott, ethereal undulations may be produced, with the consequent accumulation of ethereal polarity requisite to give sparks before contact, agreeably to the experiments of those ingenious philosophers. Hence it may be presumed, that, during intense electro-ponderable polarization, superficial ethereal waves may always be a secondary effect, although the conducting power of the reagents, requisite to the

* According to Colomb's experiments, electrical attraction and repulsion are as the squares of the distances, and the force of the inductive power of statical charges which are produced by those forces, must, of course, obey the same law. Also the spark which is the result of these powers should come under this law since it must be as the generating forces. That the inductive influence has a growth commensurate with the electrifying energy is shewn by the fact ascertained by Wheatstone, that, when a jar is discharged by two very long wires, the sparks at the two ends are simultaneous, though one produced midway by the same discharge, when tested by his ingenious process, is later in a minute degree. It follows, that the spark must be as the precursory generating induction, and, of course, obedient to the same rule.

If this calculation be correct, the intensity must be as the squares of the striking distances as indicated by sparks.

It may be urged that the striking distances as measured by the length of the sparks, is in the compound ratio of the quantity and intensity. As to the quantity, however, galvanic sources have always been treated as pre-eminent in efficacy, so that on that side there could be no disparity. Moreover I have found that in galvanic apparatus, of only one or even of two pairs, as in the calorimotors, the intensity lessened as the surfaces were enlarged. By a pair of fifty square feet, of zinc surface, a white heat could not be produced in a wire of any size, however small. The calorific power of such apparatus can only be made evident by the production of a comparatively very low temperature, in a comparatively very large mass constitution of powerful galvanic batteries, is inconsistent with that accumulation of ethereal polarity which constitutes a statical spark-giving charge.

49. In a battery consisting of one galvanic pair excited by reagents of great chemical energy, and conducting power, the calorific and electro-magnetic effects are pre-eminent; but in De Luc's electric columns, consisting of several thousands of minute pairs, feeble as to their chemical and conducting efficacy, statical phenomena predominate. This seems to be quite consistent, since on the one hand the waves of polarization must be larger and slower, as the pairs are bigger and fewer; and on the other hand smaller and more active, as the pairs are more minute and more numerous. Of course the more active the polarizing waves, the more extensive must be the sphere of their influence.

50. The extent of metallic surface being constant, the greater the number, the smaller the pairs, and the less the retro-conducting power of the exciting reagents, the greater the resemblance of the phenomena to purely statical electricity, since the ratio of the ethereal to the ethereo-ponderable waves is in that construction the greatest: vice versa, the smaller the number and the larger the pairs, or the more intense the reaction of the ponderable agents, the more the phenomena prevail, heretofore ascribed to quantity, but really due to the greater proportional association of ponderable matter in the atoms subjected to the electro-polar forces.

51. If, by means of a sieve, or any other means, iron filings be duly strewed over a paper, resting on a bar magnet, they will all become magnets, so as to arrange themselves in rows like the links of a chain. Each of the little magnets thus created, will have a polarity opposite to that of the pole of the bar magnet, which influences it at its end nearest that pole. Of course the resulting ferruginous rows formed severally by the two different poles of the bar, will have polarities as opposite as those of the said poles.

52. In an analogous mode, if two wires be made the media of a galvanic discharge, iron filings, under their influence, will receive a magnetic polarity, arranging themselves about each wire like so many tangents to as many radii proceeding from its axis. Those magnetized by one wire reacting with such as are magnetized by the other.

53. The affections of the ferruginous particles during the continuance of the current so called, are precisely like those of the same particles when under the influence of the bar magnet. The great discordancy is in the fact, that the influence of the magnet is perma-

* As the word ether is used in various senses, the syllables "*electro*" being prefixed, serve to designate that which is intended.

nent, while that of the wire is indebted for existence to a series of oppositely polarizing but transient impulses which proceed towards the middle of the circuit from each side, so as to produce reciprocal neutralization by meeting midway.

54. The effect upon the filings, as originally pointed out by Oersted, is precisely such as would arise were the ponderable matter of the wire, resolved by each impulse into innumerable little magnets, situated so as to form tangents to as many radii proceeding from the axis of the wire.

55. Independently of the filings, the wires react with each other as if their constitution, during subjection to the discharge, were such as above supposed. When the discharges through them concur in direction, they attract, because the left side of one is next the right side of the other, bringing the opposite poles of their little magnets into proximity; but when the discharge is made in opposite directions, the two right or the two left sides will be in proximity, and will, by the consequent approximation of the similar poles of the little magnets, be productive of repulsion.

56. From these last mentioned facts and considerations, it must be evident that, assuming the existence of a permanent polarity in the particles of a magnetized bar, and of transient equivalent polar affections within a galvanized wire, involves no contradiction, no absurdity, nor any thing but what is consistent with the researches and inferences of Davy, Faraday, and other eminent investigators of the phenomena of nature.

57. In order that an ethereo-ponderable particle of oxygen in any aqueous solution shall unite with an ethereo-ponderable particle of zinc in a galvanic pair, there must be a partial revolution of the whole row of ethereo-ponderable zinc atoms, with which the atom assailed is catenated by the attractions between dissimilar poles. Moreover, at the same time that the metallic atoms are thus affected, the atoms of water between the metallic surfaces must undergo a similar movement, by an analogous reaction with poles of an opposite character, and this movement must extend through the negative plate to the conductor by which it communicates with the zinc or electro-positive plate. When the circuit is open, the power of combination exercised by the zinc and oxygen is inadequate to produce this movement in the whole chain of atoms, liquid and metallic; but as it is indifferent whether any two atoms are united with each other, or with any other atoms of the same kind, the chemical force easily causes them to exchange partners, as it were, when the whole are made to form a circuitous row in due contiguity.

58. As we know that during their union with oxygen, metals give out an enormous quantity of heat and electricity, (33) it is reasonable to suppose that whenever an atom of oxygen and an atom of zinc jump into union with each other, a wave is induced in the ethereo-ponderable matter, and that this wave is sustained by the decompositions and recompositions by means of which an atom of hydrogen is evolved, at the negative plate, and, probably, enabled to assume the aëriform state. There must, at the same time, be a communication of wave polarity by its contact with the surface of the negative plate, by which the positive wave in the conductor is induced. Although the inherent polarities of the metals are not, agreeably to this view, the moving power in galvanism, yet they facilitate, and in some cases induce the exercise of that power, by enabling it to act at a distance, when otherwise it might remain inert.

59. This, I conceive, is shown in the electrolytic effect of platina upon a mixture of the gaseous elements of water; also in Groves' *gas* battery, by means of which hydrogen and oxygen gas severally react with water in syphons, so as to cause each other to condense, without any communication besides that through the platina, and an electrolytic decomposition and recomposition extending from one of the aqueous surfaces in contact with one of the gases, to the other surface in contact with the other gas.

60. The various forms of the electric spark, resulting from varying the gas, through which it may be made to pass, agreeably to the researches of Faraday, is explained by the supposition that the luminosity of the spark is partially the consequence of the polarizability of the ethereo-ponderable atoms of the gases through which the discharge is made, and consequently varies with their nature.

61. The reciprocal influence of galvano-electrical currents, and of such currents with magnetized or magnetizable masses, are well designated as *electro*-magnetic or *magneto*-electric, accordingly as the current or the magnet is the primary agent. There are, however, two other species of electro-polarity which come under the head of statical electricity. One of these Faraday illustrates by supposing three bodies, A, B and C, in proximity, but not in contact, when A, being electrified, electrifies B, and B electrifies C by induction. This Faraday calls an *action* of the particles of the bodies concerned, whereas, by his own premises, it appears to me to be merely a superficial affection of the masses or of a circumambient ethereal matter. This species of polarization, to which the insulating power of air is necessary, affects the superficies of a body only,* being displayed as well by a gilt globe of glass, as a solid globe of metal. No sensible change appears to be produced in the ponderable conducting superficies by this inductive superficial electrification of masses; and hence, probably, there is no consequent magnetism.

62. The other instance of statical polarity, is exemplified by the charge existing in a coated pane or jar. In this case, according to Fara-

* According to Colomb and others.

day, the particles of glass are thrown into a state of electro-polarity, and are, in fact, partially affected as if they belonged to a conductor; so that insulators and conductors differ only in the possession in a high degree by the one, of a susceptibility of which the other is possessed to an extent barely perceptible. The facts seem to me only to show, that either an insulator or conductor may be both affected by the same polarizing force, the transmission of which the one facilitates, the other prevents. I am under the impression that it is only by the diruptive process that electricity passes through glass; of course involving a fracture. It gets through a pane or jar, not by aid of the vitreous particles, but in despite of their opposing coherence. The glass in such cases is not liable to be fused, deflagrated, or dissipated, as conductors are. It is forced out of the way of the electrical waves, being incapable of becoming a party to them. Discharges will take place through a vacuity, rather than through the thinnest leaf of mica. But if, as Faraday has alleged, from within a glass flask hermetically sealed, an electrical charge has been found to escape, after a long time, it proves only that glass is not a perfect insulator, *not "that perfect insulation and perfect conduction are different extremes of the same property."* On the contrary, the one is founded upon a constitution competent to the propagation within it of the electro-polarizing waves, with miraculous facility, while the other is founded either on an absolute incapacity, or comparatively an infinitely small ability to be the medium of their conveyance. The one extremely retards, the other excessively expedites its passage through a void.*

63. I infer that the magnetic influence of electricity, is always due to the electro-polarization of ponderable matter, in consequence of the incorporation with it of the polarizable electro-ether,* thus constituting, as above stated, ethereo-ponderable atoms.

64. Reference has been made to two modes of electrical conduction, in one of which the efficacy is as the surface; in the other, as the area of a section of the conductor. Although glass be substantially a non-conductor, the power of the surface of glass when moistened, or gilt, to discharge statical electricity is enormous. It has been generally considered, that as a protection against lightning, the same weight of metal employed as a pipe, would be more efficacious than in the usual solid form of a lightning rod: yet this law does not hold good with respect to galvanic discharges, which are not expedited by a mere extension of conducting surface. Independently of the augmentation of conducting power, consequent to the cooling influence of radiation, and contact with the air, which, according to Davy, promotes conduction, a metallic ribbon does not

* By a void, I mean a Torricillian vacuum. The omnipresence of the electro-ether must render the existence of a perfect void impossible.

conduct a galvanic current better than a wire of similar weight and length.[1]

65. The sectional area of a conductor remaining the same, in proportion as any *statical* accumulation which it may discharge is greater, the effects are less superficial; and the ethereo-ponderable atoms are affected more analogously to those exposed to galvanic discharges. It is in this way that the discharge of a Leyden jar imparts magnetic polarization. Thus, on the one hand, the electro-ethereal matter being polarized and greatly condensed, combines with and communicates polarization, and consequently magnetism, to ethereo-ponderable bodies; while, on the other hand, these, when polarized by galvanic reaction, and thus rendered magnetic, communicate polarity to the electro-ether. Hence statical electricity, when produced by galvanism, and magnetism, when produced by statical electricity, are secondary effects.

66. Where a wire is of such dimensions, in proportion to the charge, as to be heated, ignited, or dispersed by statical electricity, there seems to be a transitory concentration of the electric power, which transforms the nature of the reaction, and an internal wave of electro-ponderable polarization, similar to those of galvano-electricity, is the consequence.

67. As above observed, (34,) the current produced by the magneto-electric machine, has all the attributes of the galvano-electric current; yet this is altogether a secondary effect of the changes of polarity in a keeper, acting upon a wire solely by dynamic induction. But if, by mere external influence, the machine above mentioned can produce within a circuit a current such as above described, is it unreasonable to suppose that the common machine, when it acts upon a circuit, may put into activity the matter existing therein, so as to produce waves of polarization, having the power of those usually ascribed to a galvano-electrical current?

68. It has been shewn that both reason, and the researches and suggestions of Faraday, warrant the inference that ponderable atoms, in solids and liquids, may be considered as swimming in an enormous quantity of condensed imponderable matter, in which all the particles, whether ponderable or imponderable, are, in their natural state, held in a certain relative position due to the reciprocal attraction of their dissimilar poles. An electrified body differs from one in its ordinary state, in having the polarity of the ponderable or imponderable matter existing within, or about it, one or *both* deranged.

69. In statical excitement the affection is superficial as respects the ponderable bodies concerned, while in dynamic excitement the polarities of the whole mass are deranged oppositely at opposite ends of the electrified mass; so that the oppositely disturbing impulses, proceeding from the poles of the disturbing apparatus, neutralize each other intermediately. Supposing the ponderable as well as the imponderable matter in a perfect conductor, to be susceptible of the polar derangement, of which an electrified state is thus represented to consist, non-conductors to be insusceptible of such polar derangement, imperfect conductors may have a constitution intermediate between metals and electrics. When an electrical discharge is made through any space devoid of air or other matter, it must then find its way solely by the polarization of rare imponderable matter existing therein; and consequently its corruscations should be proportionably more diffuse, which is actually found to be true; but when gaseous ethereo-ponderable atoms intervene, they enable competent waves to exist within a narrower channel, and to attain a greater intensity. I consider all bodies as insulators which cause discharges through them to be more difficult than through a vacuum, and which, consequently, cannot be made to convey the waves from one body to another when the transfer would not take place in their absence. This furnishes a good mean of discrimination between insulators and conductors, the criterion being that a discharge ensues more readily as there is more of the one and less of the other in the way. Even when a bell wire has been dissipated by lightning, it has been found to facilitate and determine the path of the discharge. Both in the case of disruptive discharge through air, producing a spark, or of a deflagrating discharge through wire, causing its explosion, there is a dispersion of intervening ponderable particles; and yet there is this manifest discordancy, that in the one case the undulatory process of transfer is assisted, in the other resisted. The waves follow the metallic filament with an intense attraction, while they strive to get out of the way of those formed by the aëriform matter, as if repelled. Hence the term diruptive, from dirumpo, to break through, was happily employed by Faraday to designate spark discharges. The zigzag form of the diruptive spark, shows that there is a tendency in the aëriform particles to turn the waves out of that straight course, which, if unresisted or facilitated, they would naturally pursue. On the one hand the aërial filaments being unsuitable for the conveyanee of the electric waves, they are forced by them out of the normal path, first in one direction, then in another; while, on the other hand, the finest metallic filament furnishes a channel for the electric waves, so favorable that this channel is pursued, although the consequent polarization of the conducting particles be so intense as to make them fly asunder with explosive violence.

70. When a small image, of which the scalp has been abundantly furnished with long hair, is electrified, the hairy filaments extend themselves and move apart, as if actuated by a repulsive power: also when iron filings are so managed as to obey the influence of the poles of a powerful magnet, they arrange themselves in a manner resembling that of the electrified hair. There is, moreover, this additional analogy, that there is an attraction between two portions of hair differently electrified, like that which arises between filings differently magnetized.—Yet the properties of the electrified hair and magnetized filings are, in some respects, utterly dissimilar. A conducting communication between differently electrified portions of hair would entirely neutralize the respective electrical states; so that all the electrical phenomena displayed by them would cease. Yet such a communication made between the poles, exciting the filings, by any non-magnetic conductor, does not in the slightest degree lessen their polar affections and consequent power of reciprocal influence. Upon the electrified hair, the proximity or the contact of a steel magnet has no more effect than would result under like circumstances from any other metallic mass similarly employed; but by the approximation, and still more, the contact, of such a magnet, the affection of the filings may be enhanced, lessened, or nullified, according to the mode of its employment. In the case of the hair, the affection is superficial, and the requisite charging power must be in proportion to the extent of surface. In the case of the magnetized ferruginous particles, it is the mass which is affected, and, cæteris paribus, the more metal, the greater the capacity for magnetic power. In the instance of the electrified hair, as in every other of statical excitement, the electrical power resides in imponderable ethereo-electric atmospheres which adhere superficially to the masses, being liable to be unequally distributed upon them in opposite states of polarity, consequent to a superficial polarization of the exciting or excited ponderable masses; but in the instance of bodies permanently magnetic, or those rendered transiently magnetic by galvanic influence, the ethereo-electric matter and the ponderable atoms are inferred to be in a state of combination, forming ethereo-ponderable atoms; so that both may become parties to the movements and affections of which the positive and negative waves consist. Since the electro-polarization, when carried to a certain degree of intensity, produces ignition, at other degrees deflagration, decomposition, or dispersion of conductors, it may be conceived, that in discharges too feeble to produce these consequences, there is still proportionably an affection of the ponderable matter.

71. I infer that the difference between the phenomena of statical electricity and those of galvanism does not arise from a difference in quantity and intensity, but from the greater or less extent in which the polarity of the ponderable atoms of the masses affected are deranged. All magneto-polar charges, I infer to be attended by an affection of ponderable particles; and the reason why the most intense statical charge does not affect a galvanometer, is that it is only when oppositely excited bodies are neutralized by the interposition of a conductor, as during a discharge, that ethereo-ponderable particles are sufficiently polarized to enable them to act upon others in their vicinity, so as to produce a polar affection the opposite of their own. In this way dynamic induction is consistently explained, by supposing that the waves of polarization, in passing along one conductor, produce, pari passu, the opposite polarization in the proximate part of any neighboring conductor suitably constituted, situated and arranged to allow it to form a part of a circuit. A magnetized bar, so contrived as to rotate easily upon its axis, turns to the right or to the left, when made the channel of two opposite galvanic discharges, accordingly as the connections with the battery may be made. This may be explained by supposing that the opposite undulations (consistently with Oersted's idea) moving spirally, one to the right the other to the left, react with the polarized ethereo-ponderable particles of the magnetized bar, so as to produce revolutionary impulses.

72. It is only during the state of the incessant generation and destruction of what has been called the two electricities, that the circuit, which is the channel for the passage of the polarizing waves, is endowed with electro-magnetic powers. It was no doubt, in obedience to a perception of this fact, that Oersted ascribed the magnetism of a galvanised wire to a conflict of the electricities. Undoubtedly that state of a conductor in which, by being a part of an electrical circuit, it becomes enabled to display electro-magnetic powers, is so far a conflict of the two electricities, as the affections of matter which are denominated electrical consist of two opposite polar forces, proceeding, agreeably to the language of Faraday, in opposite directions from each side of the source, and conflicting with each other so as to be productive of reciprocal annihilation.

73. That a corpuscular change in conductors is concomitant with their subjection to, or emancipation from, a galvanic current, is proved by an experiment of Henry's, which he afforded me an opportunity, on one occasion, of witnessing. I allude to the fact that sound is produced whenever the circuit is suddenly made or suddenly ruptured. By I. P. Marrian it has been observed, that a similar result takes place during the magnetization or demagnetization of iron rods, by the alternate establishment or arrestation of galvanic discharges through wires coiled about them so as to convert each into an electro-magnet. Mr. Marrian represents the sound as resembling that produced by striking a rod upon one of its ends.* †

74. Thus it appears that there is an analogy between the state of matter which involves permanent magnetism and that which constitutes a galvanic current, so far as this; that either by one or the other, during either its access or cessation, an affection of the ponderable particles concerned ensues, sufficient to produce sound.

75. Although waves originally purely ethereal, as when generated by a discharge from a conductor or Leyden jar, are prone to pass superficially; yet as the surface of the conducting wire becomes less, the reaction between the ethereal and ethereo-ponderable matter becomes greater; so that finally deflagration may be produced. Hence the inferiority of statical discharges in producing electro-magnetic power is explained by the fact, that this power being in the compound ratio of the quantity of matter affected and the intensity of the affection, neither condition can be augmented without a diminution of the other. In order to produce the intensity requisite to the polarization of the ponderable particles, we must lessen the number of these subjected to the discharge, and, of course, the numerical force which can be brought into the electro-magnetic battle-field.

*Summary.*

From the facts and reasoning which have been above stated, it is presumed that the following deductions may be considered as highly probable, if not altogether susceptible of demonstration.

The theories of Franklin, Dufay and Ampere, are irreconcilable with facts on all sides admitted.

A charge of statical electricity, or that species of electric excitement which is produced by friction, is not due to any accumulation, nor any deficiency either of one or of two fluids, but to the opposite polarities induced in imponderable ethereal matter existing throughout space however otherwise void, and likewise condensed more or less within ponderable bodies, so as to enter into combination with their particles, forming atoms which may be designated as ethereo-ponderable.

Statical charges of electricity seek the surfaces of bodies to which they may be imparted, without sensibly affecting the ethereo-ponderable matter of which they consist.

When surfaces thus oppositely charged, or, in other words, having about them oppositely polarized ethereal atmospheres,

* Agreeably to recent experiments of Faraday, the particles of a glass prism may be so influenced by an electro-magnet as to affect the passage of polarized light.

† L. and E. Phil. Mag. and Jour., vol. 45, p. 383, 1844.

are made to communicate, no current takes place, nor any transfer of the polarized matter: yet any conductor touching both atmospheres, furnishes a channel through which the opposite polarities are reciprocally neutralized by being communicated wave-like to an intermediate point.

Galvano-electric discharges are likewise effected by waves of opposite polarization, without any flow of matter meriting to be called a current.

But such waves are not propagated superficially through the purely ethereal medium; they occur in masses formed both of the ethereal and ponderable matter. If the generation of statical electricity, capable of influencing the gold leaf electrometer, indicate that there are some purely ethereal waves induced by the galvano-electric reaction, such waves arise from the inductive influence of those created in the ethereo-ponderable matter.

Magnetism, when stationary, as in magnetic needles and other permanent magnets, appears to be owing to an enduring polarization of the ethereo-ponderable atoms.

The magnetism transiently exhibited by a galvanized wire, is due to oppositely polarizing impulses, severally proceeding wave-like to an intermediate part of the circuit where reciprocal neutralization ensues.

When magnetism is produced by a statical discharge operating upon a conducting wire, it must be deemed a secondary effect, arising from the polarizing influence of the ethereal waves upon the ethereo-ponderable atoms of the wire.

Such waves pass superficially in preference; but when the wire is comparatively small, the reaction between the waves and ethereo-ponderable atoms becomes sufficiently powerful to polarize them, and thus render them competent, for an extremely minute period of time, to produce all the affections of a galvano-electric current, whether of ignition, of electrolysis, or magnetization. Thus, as the ethereo-ponderable waves produce such as are purely ethereal, so purely ethereal waves may produce such as are ethereo-ponderable.

Polarization of hair upon electrified scalps is supposed to be due to purely ethereal polarity, while that of iron filings, by a magnet or galvanized wire, is conceived to be owing to ethereo-ponderable polarity.

